# PERCEIVED AGE DISCRIMINATION IN THE SECOND HALF OF LIFE: AN EXAMINATION OF AGE, PERIOD, AND COHORT EFFECTS

**DOI:** 10.1093/geroni/igad104.1211

**Published:** 2023-12-21

**Authors:** Liat Ayalon, Octavio Bramajo

**Affiliations:** Bar Ilan University, Ramat Gan, HaMerkaz, Israel; Autonomous University of Barcelona, Cerdanyola del Valles, Catalonia, Spain

## Abstract

The present study aimed to examine variations in perceived age discrimination in the second half of life. Perceived age discrimination is different from objective age discrimination as it depends on respondents’ recognition, acknowledgement, and willingness to report the discriminatory event. We adopt a comprehensive approach which examines whether perceived age discrimination varies by age (chronological time from birth), period (when data were collected), or cohort (a group of people with shared life events experienced at a similar age) across gender and ethnic origin. Relying on psychosocial data from Health and Retirement Survey between 2006 and 2018. Our findings show that perceived age discrimination increases with age. In addition, older cohorts of women are more likely to report perceived age discrimination, whereas White women born around 1940 are less likely to report perceived age discrimination. White men, in contrast show a slight increase in perceived discrimination. Results are discussed from an intersectional perspective, which considers societal life course events.

